# Potency testing for a recombinant protein vaccine early in clinical development: Lessons from the *Schistosoma mansoni* Tetraspanin 2 vaccine

**DOI:** 10.1016/j.jvacx.2021.100100

**Published:** 2021-06-06

**Authors:** Guangzhao Li, Lara Hoeweler, Brian Keegan, Jin Peng, Larissa Scholte, Peter Hotez, Maria Elena Bottazzi, David Diemert, Jeffrey Bethony

**Affiliations:** a Department of Microbiology, Immunology and Tropical Medicine, The George Washington University, Washington DC, USA; b Texas Children's Hospital Center for Vaccine Development, USA; c Department of Pediatrics, National School of Tropical Medicine, Baylor College of Medicine, Houston, TX, USA; d Department of Medicine, The George Washington University, Washington DC, USA

**Keywords:** Vaccine, Potency, Compliance approach, Least-squares regression, Control charting, Bootstrap modeling

## Abstract

**Introduction:**

As a primary stability-indicating parameter, potency should be strategically evaluated during each phase of vaccine development. Herein, we present potency testing during the early clinical development of the *Schistosoma mansoni* (*Sm*) Tetraspanin-2 vaccine formulated on Alhydrogel *(Sm*-TSP-2/Al). As *Sm*-TSP-2/Al does not induce sterilizing immunity against its target pathogen (*Sm*) in animal models, potency is measured by “*serological substitution”,* a method that can add significant variation to the potency metric, especially when used in a compliance (or ‘*single data point’*) approach.

**Methods:**

Potency data were analyzed using the compliance approach to determine if two clinical lots of *Sm*-TSP-2/Al retained potency over 84 and 36 months post-release, respectively. These same data were also analyzed by: i) least-squares regression with a joinpoint regression analysis; ii) control charting of stability slopes; and iii) bootstrap modeling. Nested-regression and bootstrapping were used to compare the potency of the first (#11-69F-003) and second (#1975) clinical lots of *Sm*-TSP-2/Al.

**Results:**

Despite significant variability in the immune assay, both clinical lots of *Sm*-TSP-2/Al remained potent for 84 and 36 months, respectively, in all four statistical approaches. The first lot of *Sm*-TSP-2/Al showed a gain in potency starting at 36 months post-release as captured by joinpoint regression. The two clinical lots of *Sm*-TSP-2/Al had comparable long-term potency.

**Conclusion:**

While a compliance approach can monitor the long-term stability of *Sm*-TSP-2/Al, it risks putting this critical stability-indicating parameter out of specification with each time point tested due to statistical multiplicity. Alternative statistical methods, such as joinpoint regression or bootstrapping, do not have this limitation and offer even more precise estimations of potency, with the added benefit of also providing predictive analytics. Nested regression and bootstrapping were shown to be a viable alternatives for lot-to-lot comparisons of the stability of *Sm*-TSP-2/Al. Instructions for implementing both these potency testing approaches are provided.

## 1 Introduction

A stability testing program is a critical component in vaccine development, with potency considered the primary “*stability-indicating parameter*” [1]. Potency testing should occur over the lifespan of a vaccine as its biological activity can alter significantly during storage, with a “loss” or “gain” of potency critical for decisions concerning the ongoing administration of the product [2]. The design of these studies should strategically address the objective of each phase of vaccine development [3]. For example, during early clinical development, potency testing should be designed to determine an acceptable potency range immediately after manufacture and assess the product’s safety; however, in later vaccine development, potency testing should be designed to determine the vaccine’s shelf life or the biological activity of newly manufactured lots of the vaccine.

When potency is measured using a traditional “animal-challenge” model, as is the case for most attenuated viral and bacterial vaccines [4], the primary stability-indicating parameter is the vaccine’s continued ability to protect immunized animals against challenge from the target pathogen [5] as assessed by the median (or *mean*) lethal dose (LD_50_). Alternatively, for vaccines that do not induce sterilizing immunity against their target pathogens (e.g., malaria [6], schistosomes [7], and hookworms [7]), potency can be estimated by measuring an immune response using a *“serological substitution*” method [8]. In this method, potency refers to the product’s ability to generate an antibody response in animals as assessed by the median effective dose (ED_50_) or the lowest dose of the vaccine that induces antibodies in 50% of the animals in a dose group [9]. While serological substitution has the benefit of measuring a hypothesized mechanism of action for many recombinant protein vaccines (e.g., neutralizing antibodies), it also has the drawback of incorporating significant assay variation into the potency metric, which is especially problematic when potency is measured in a compliance or “*single data point*” testing approach [10].

In a compliance approach, potency is measured at “independent” time points post-release to determine if the biological activity remains within an acceptable range set at the product’s lot release or immediately after current Good Manufacturing Practice (cGMP) production [10]. Several problems attend conventional compliance approaches to potency testing. The first is that, early in clinical development, due to the product’s recent manufacture, the acceptable potency range is often derived from sparse empirical data. The second is that, when measured using *serological substitution*, the potency metric is a composite of any real change in the product’s biological activity and the variation inherent in the immune assay [10]. Finally, in the compliance approach, each post-release time point is considered independently; therefore, each additional time point tested increases the probability of potency being out of specification (OOS) due to assay variation alone (“*statistical multiplicity*”) [10]. These limitations have led to the assertion that a compliance approach “*discourage[s] potency data collection*” [10].

Herein, we present a potency testing strategy for the early clinical development of the recombinant *Schistosoma mansoni* (*Sm*) Tetraspanin-2 vaccine formulated on Alhydrogel *(Sm*-TSP-2/Al), which is being developed for use in children and adults to prevent morbidity due to chronic intestinal/hepatic schistosomiasis. The first (#11-69F-003) and second (#1975) clinical lots of *Sm*-TSP-2/Al, manufactured four years apart to meet product needs during early clinical testing, were tested for potency over their first 84 and 36 months, respectively, after cGMP production. As *Sm*-TSP-2/Al does not induce sterilizing immunity against its target pathogen (*Sm*) in an animal model, potency was estimated by *serological substitution* using a compliance approach and the *relative potency* (RP) metric, as has been done for other recombinant protein vaccines developed by our group [11], [12]. Three alternatives to the *compliance approach* for potency testing were then applied using the same potency dataset: (i) a least-squares regression fitted to a first-order linear decay model [10] followed by joinpoint regression to identify segments of time when potency deviated from first-order decay; (b) control charting of stability slopes, followed by assessment of their conformity to the Westgard rules of quality control; and (c) bootstrap models of potency over time and by cGMP manufactured lot, including a bootstrap simulation of a sub-potent lot of *Sm*-TSP-2/Al. It is hoped that this compendium of novel statistical approaches to potency testing offers new ways of thinking about this critical *stability-indicating parameter* early in clinical development of investigational vaccines.

## 2 Materials and methods

### 2.1 *Sm*-TSP-2/Al potency testing approach

Manufacture of clinical lots of the recombinant *Schistosoma mansoni* tetraspanin-2 protein formulated on Alhydrogel® (*Sm*-TSP-2/Al) is described in detail in Supplementary Text 1. The design of the potency tests conducted at each time point is shown in Table 1. The potency testing approach for this vaccine has been previously described by us in several manuscripts [12], [7], [13] and is detailed again in Supplementary Text 2. Fig. S1 shows the standard calibration curves (SCCs), used for potency testing of both clinical lots, including the fully specified logit-log model that linearizes the sigmoidal shape of the standard curves to estimate the statistical similarity of the SCCs for each independent potency time point post-release. Fig. S2 graphically represents the estimation of the reactivity threshold (RT) and a limit of quantitation (LOQ) used for the quantal response to estimate potency for *Sm*-TSP-2/Al. All potency data are deposited in the Mendeley Data (doi: https://doi.org//10.17632/3r2bfsjyzz.1).

**Table 1:** Design of the *Sm*-TSP-2/Al mouse potency assay in which groups of 10 BALB/c mice are vaccinated by the doses of adjuvant, Clinical Drug Substance, and Clinical Drug Product below to estimate the median Effective Dose or ED_50_ and relative potency at release and pre-defined post-release timepoints.

Group	Formulation	Dose (μg)^a^		Volume (mL)
		*Sm*-TSP-2	Alhydrogel®	
**1**	Adjuvant	–	400	0.500
**2**	Clinical Drug Substance	50	–	0.023
**3**	Clinical Drug Product	50	400	0.500
**4**	Clinical Drug Product	28.57	228.56	0.286
**5**	Clinical Drug Product	16.33	130.64	0.163
**6**	Clinical Drug Product	9.33	74.64	0.093
**7**	Clinical Drug Product	5.33	42.64	0.053
**8**	Clinical Drug Product	3.05	24.4	0.031
**9**	Clinical Drug Product	1.74	13.92	0.017
**10**	Clinical Drug Product	0.99	7.92	0.010
**11**	Immunizability Control^b^	9.33	74.64	0.093

a The symbol “--” means not applicable.

b The term “immunizability control” refers to vaccination using Formulated Reference Standard (Reference Standard drug substance formulated with Alhydrogel®) and is used to determine variation in the immune response from different “lots” of animals sent to the vivarium. No comparisons are made to the antibody values generated by this group with the clinical drug product.

### 2.2 Compliance approach

The long-term assessment of stability of both clinical vaccine lots was estimated by a Relative Potency (RP) metric in a compliance approach, with RP estimated at pre-defined independent time points post-release using a parallel-line assay model [12], [13]. After parallelism and linearity of dose–response curves were established for potency at release and potency at each post-release time point, the RP was then calculated as follows:

$$M_{T}=\frac{a_{P}-a_{R}}{b}$$

where *M*_T_ is ln (potency ratio), $a_{P}$ is the intercept of the dose–response curve at a particular post-release time point, $a_{R}$ is the intercept of the dose–response curve at release, and *b* is the common slope. Additional information about RP derivation and application is available in Supplementary Text 3. The current specification is to reject a vaccine lot when the upper 95% confidence limit of the RP is below 0.5 [12].

### 2.3 Immune assay variation and the role of statistical multiplicity in the compliance model

To estimate immune assay variation**,** a positive control (PC) consisting of the qualified Standard Reference Serum of murine IgG against *Sm*-TSP-2 was added at a 1:6000 dilution to each microtiter plate. The variance of the PCs among potency runs from both lots was used as an index for inter-run assay variation. The association between the mean PC for each run and the ED_50_ was determined by linear regression analysis, and the effect of assay variance on potency was expressed as *R^2^*. The probability of out-of-specification was then calculated for each time point.

### 2.4 Nested regression for lot-to-lot variation

Potency comparisons between the first and second manufactured lots of *Sm*-TSP-2/Al at release and at common pre-defined post-release timepoints were made using nested models [14]. A binary *probit* regression model between two lots was specified as:

$$P=Pr\left(Response\right)=F\left(\beta_{0}+\beta_{1}D+\beta_{2}I+\beta_{3}I*D\right)$$

where *P* is the seropositivity proportion, *D* is the log10-converted dose of *Sm*-TSP-2, and *I* is an indicator discriminating test lot (i.e., *I* = 0 for the first lot and *I* = 1 for the second lot). Thus, β_0_ represents the intercept, and β_1_ represents the slope of the first lot; and (β_0_ + β_2_) represents the intercept, and (β_1_ + β_3_) represents the slope of the second lot. The likelihood ratio test (LRT) was used to evaluate the hypothesis that β_2_ = β_3_ = 0, i.e., the two dose–response relationships were identical. SAS coding is provided in Supplementary Text 4.

### 2.5 Least-squares regression analysis followed by joinpoint regression

In the context of a constant trend, the relationship between ED_50_ and post-release time point was modeled using least-squares linear regression on a logarithmic scale:

$$lnED_{50}\left(t\right)=lnED_{50}\left(0\right)-kt$$

Derivation and application of this first-order decay kinetics model can be found in Supplementary Text 5a. When variation is present in the relationship between the time point and ED_50_, a joinpoint regression (also referred to as linear spine regression) was employed to model this change in the potency trend. The relationship between ED_50_ and months post-release was modeled as:

$$lnED_{50}\left(t\right)=lnED_{50}\left(0\right)-kt+\alpha {\left(t-\tau \right)}^{+}$$

where τ is the break-point and,

$${\left(t-\tau \right)}^{+}=\left\{\begin{array}{c} t-\tau ,if\;months\;post-release>\tau \\ 0,if\;months\;post-release\leq \tau . \end{array}\right)$$

Step-by-step instruction for the application of the maximum-likelihood (ML) estimation approach [15] are in Supplementary Text 5b. Joinpoint regression model fitting and break-point estimation were performed in R using the package “*segmented*” with the *segmented* function. The 95% confidence interval around the regression line was generated, and the earliest time at which the 95% confidence limit intersects the proposed acceptance criterion predicts the vaccine’s stability.

### 2.6 Control charting of stability slopes with conformity to Westgard rules of quality control

Using *probit* analysis, for a given Dose level, the probability *P* of a positive response is modeled as:

$$P=Pr\left(Response\right)=F(b_{0}+b_{1}\times log_{10}\left(Dose\right))$$

The slope *b_1_* represents the rate of change in the probability of seroconversion for a small amount of change in logarithm-transformed dosage level, i.e., the higher the slope, the more likely the mouse undergoes seroconversion given the same injected dose. Estimates of the stability slopes for 16 time points of the first lot (#11-69F-003) and 8 of the second lot (#1975) were presented in a Levy-Jennings control chart, with the potency metric expected to conform to the Westgard Multirule Quality Control standard [16]. A vaccine lot was considered OOS, and therefore rejected, when the stability slope *b_1_* exceeded the mean minus three standard deviations (3 s). Step-by-step instruction on the application of the control chart utilizing the Westgard rules can be found in Supplementary Text 6.

### 2.7 Bootstrap estimation of potency by time, lot-to-lot comparison, and modeling a sub-potent lot

Step-by-step instruction on the application of the bootstrapping model utilizing the *boot*() function of the R package “*boot*” [17], [18] can be found in Supplementary Text 7a for stability evaluation and Supplementary Text 7b for lot-to-lot comparison. For stability evaluation, the resulting empirical distributions of the regression stability slope *b_1_* were utilized to demonstrate the differences in potency between a “current” time point response and a “cumulative” time point response. “Current” refers to the bootstrapped stability slope generated using the quantal response data at time point *n*, and “cumulative” refers to the bootstrapped stability slope generated using the pooled quantal response data until time point *n*. A vaccine lot was considered OOS if the upper limit of the bootstrapped confidence interval of the “current” potency test was below the lower limit of the bootstrapped confidence interval of the “cumulative” potency test. For lot-to-lot comparison, the resulting empirical distributions of regression stability slope *b_1_* were utilized to demonstrate the differences in potency between the first (#11-69F-003) and second (#1975) clinical lots at each time point. Future stability slopes that fell below the estimate of stability slope of potential sub-potent lot were flagged as OOS, and the frequency of testing was increased to define the exact time point at which the OOS occurred.

## 3 Results

### 3.1 Relative potency of *Sm*-TSP-2/Al as estimated in a compliance model

**Fig. 1:**
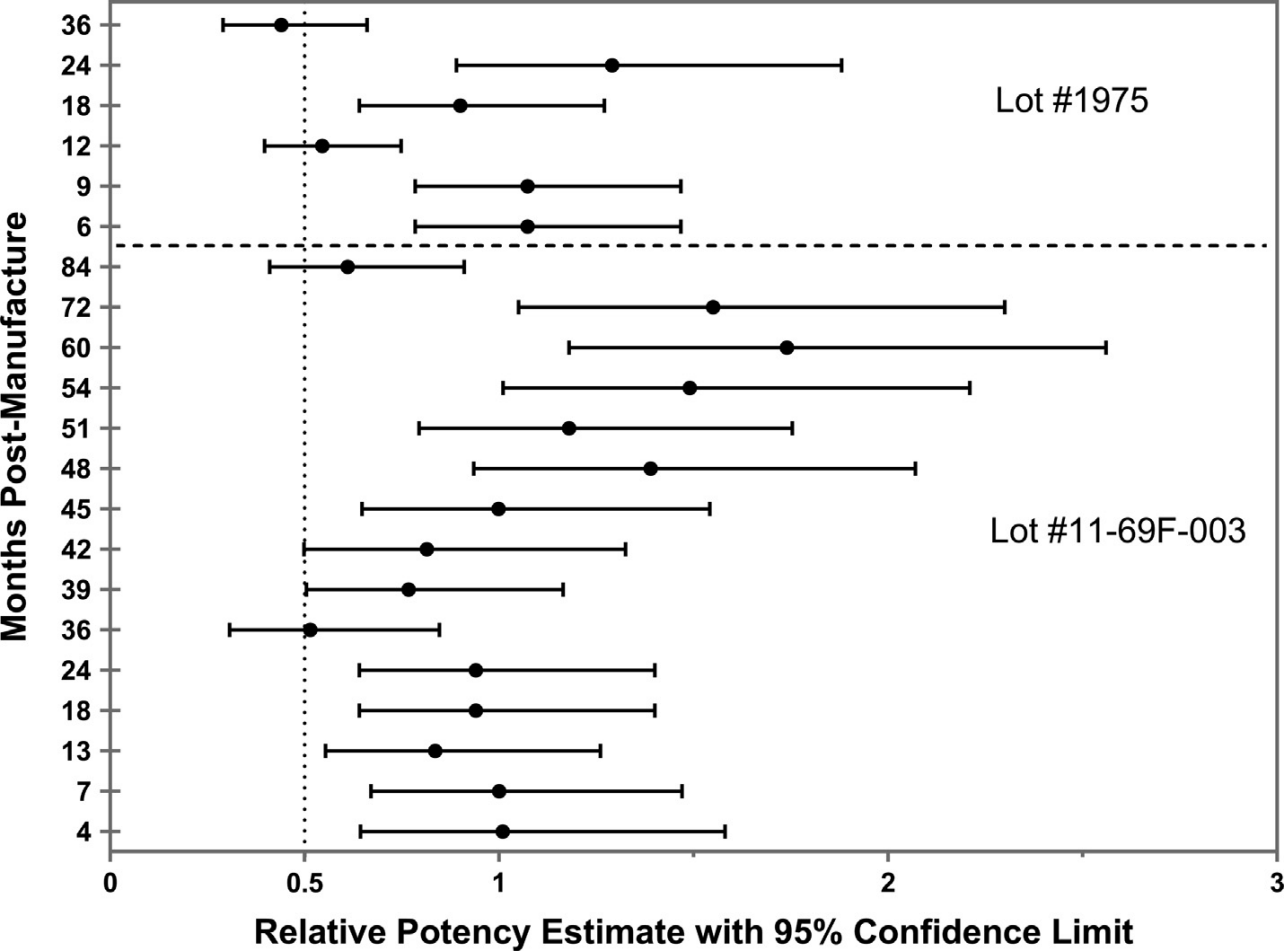
**Estimates of relative potency using a compliance model at 84 and 36 months post-release for the first (#11-69F-003) and second (#1975) clinical lots, respectively.***Sm*-TSP-2 vaccine, which is at a concentration of 0.1 mg/mL *Sm*-TSP-2 with 0.8 mg/mL of Alhydrogel® in a sucrose/imidazole/Phosphate buffer (15% sucrose, 10 mM imidazole, 2 mM Phosphate, pH 7.4), was manufactured under current Good Manufacturing Practice (cGMP) conditions and stored in temperature-monitored refrigerators at 2–8 °C. The X-axis represents the relative potency estimates in black solid circle and its 95% confidence limits in black error bars. The Y-axis represents the testing time points in months post-manufacture. The vertical dotted line at 0.5 represents the specification for acceptance that the upper 95% confidence limit of the RP should not be less than 0.50.

Supplementary Table 2 shows the seropositivity of test samples for both vaccine lots, whereas Table 2 shows the ED_50_ and RP results. Additional specifications of the lots (color and appearance, pH, protein content, percentage adsorbed protein, sterility and identity (SDS-PAGE)) are shown in Supplementary Table 4 and values for those specifications remain stable during the testing period for both lots. The ED_50_ was 4.82 µg (95% CI: 3.52–6.71) for the first lot (#11-69F-003), and 3.83 µg (95% CI: 2.92–4.99) for the second lot (#1975), at the release time point, from which the RP specifications of the respective manufactured lots were derived for subsequent stability testing time points. For the second lot of *Sm*-TSP-2/Al, month 3 was used as the release time-point instead of month 0, as there was a departure from linearity based on the response pattern at month 0, which was OOS for release. The RPs of the clinical vaccine lots were estimated at post-release time points for both lots, as shown in Fig. 1.

**Table 2:** The median Effective Dose (ED_50_) and Relative Potency at release and at pre-defined post-release timepoints of two lots in a long-term stability evaluation program.

Potency Time Point by Month																
Aeras Lot #11-69f-003																
Potency Metric	0^a^	4	7	13	18	24	36	39	42	45	48	51	54	60	72	84
**ED_50_**^b^	4.82	4.78	4.73	5.75	4.98	4.98	9.39	6.24	5.78	4.82	3.45	4.03	3.15	2.93	3.1	7.8
**(95% CL)**^c^	(3.52, 6.71)	(2.38, 9.13)	(3.77, 6.02)	(4.3, 7.59)	(3.97, 6.37)	(3.97, 6.37)	(6.17, 14.36)	(4.67, 8.4)	N/A	(3.49, 6.59)	(2.67, 4.4)	(3.14, 5.17)	(2.51, 4.06)	N/A	(2.41, 3.89)	(5.98, 10.29)
**RP**	–	1.01	1	0.84	0.94	0.94	0.51	0.77	0.81	1	1.39	1.18	1.49	1.74	1.55	0.61
**(95% CL)**	–	(0.64, 1.58)	(0.67, 1.47)	(0.55, 1.26)	(0.64, 1.4)	(0.64, 1.4)	(0.31, 0.85)	(0.5, 1.16)	(0.5, 1.33)	(0.65, 1.54)	(0.93, 2.07)	(0.79, 1.75)	(1.01, 2.21)	(1.18, 2.56)	(1.05, 2.30)	(0.41, 0.91)

WRAIR Lot #1975																
**Potency**																

**Metric**	**0**	**3**^a^	**6**	**9**	**12**	**18**	**24**	**36**								

**ED_50_**	4.05	3.83	3.24	3.24	7.05	4.24	2.96	8.59								
**(95% CL)**	N/A	(2.92, 4.99)	N/A	N/A	(5.77, 8.61)	(3.30, 5.49)	(2.23, 3.98)	(4.03, 17.36)								
**RP**	–	–	1.073	1.073	0.545	0.9	1.29	0.44								
**(95% CL)**	–	–	(0.78, 1.47)	(0.78, 1.47)	(0.40, 0.75)	(0.64, 1.27)	(0.89, 1.88)	(0.29, 0.66)								

a Potency at “lot release” based on which relative potency is calculated for subsequent time points.

b The theoretical dose in µg that would produce seroconversion in 50% of the mice.

c 95% Confidence Limits.

### 3.2 Immune assay variation and statistical multiplicity

The simple linear regression gave an R-square of 8.5% when PC was the predictor and ED_50_ was the response, implying that approximately 8.5% of the variability in the potency metric or ED_50_ was in fact explained by variation in the assay. i.e., when evaluating the stability of *Sm*-TSP-2/Al at a single time point, there is an 8.5% chance of incorrectly rejecting the lot, if the lot is in fact stays within specification. The probability of falsely rejecting a lot at one or more time points becomes much higher as testing points increase, and this probability can be calculated as 1-(1–8.5%)^n^, where n is the number of testing points. Hence, at month 3, after testing two timepoints, the probability that one or two measurements would be OOS is 16.3%, indicating the probability of rejecting at least one timepoints by chance is 16.3%, even if the lot stays within specification. This probability increases even more if more testing is performed as shown in Supplementary Table 1.

### 3.3 Nested regression using a compliance model for potency comparison of the first (#11-69F-003) and second (#1975) clinical lots of *Sm*-TSP-2/Al

**Fig. 2:**
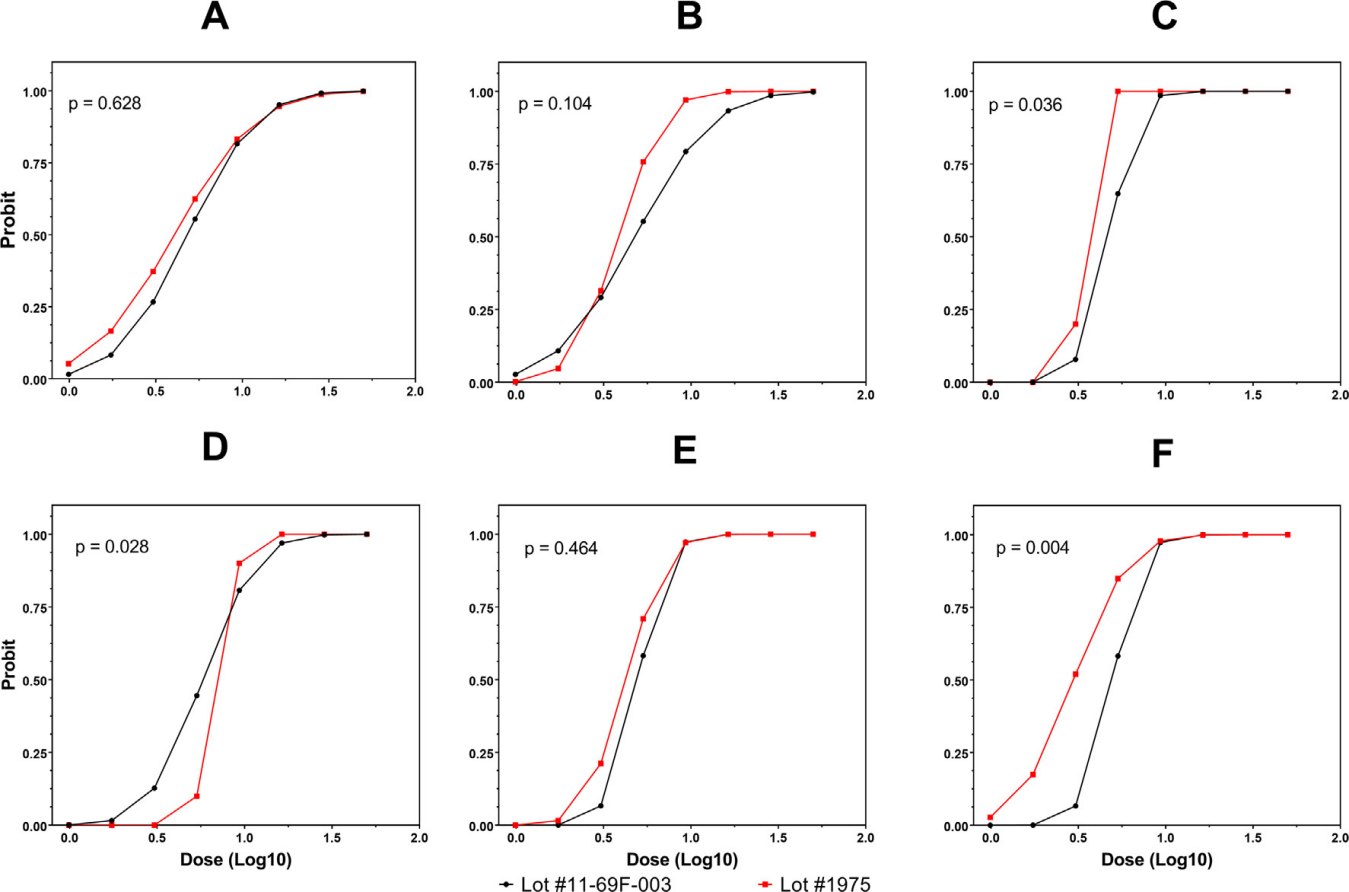
**Nested regression using a compliance model comparing the potency of the first (#11-69F-003) and second (#1975) clinical lots across single time points**. The X-axis represents the log-transformed (base 10) dose level in µg and the Y-axis represents the estimated probability of seroconversion. The dots show the predicted seropositivity probabilities at each dose level using *probit* regression. Panels A, B, C, D, E and F show results at release, 3rd, 6th, 12th, 18th and 24th month, respectively.

Scatterplots of predicted seroconversion probability and dose level at release and at months 3, 6, 12, 18, and 24 for the first and second manufactured lots of *Sm*-TSP-2/Al are presented in Fig. 2. A likelihood ratio test indicated that β_2_ = β_3_ = 0 could not be rejected at release, month 3 or month 18 (*p* > 0.05), which suggested that the dose–response relationships were the same for both lots. However, the hypothesis β_2_ = β_3_ = 0 was rejected during an examination of the data at months 6, 12, and 24 (*p* < 0.05), indicating that the dose–response relationships were not equivalent between the two lots at these time points. Test results and associated *p*-values are presented in Supplementary Table 3.

### 3.4 Least squares regression followed by joinpoint regression modelling

**Fig. 3:**
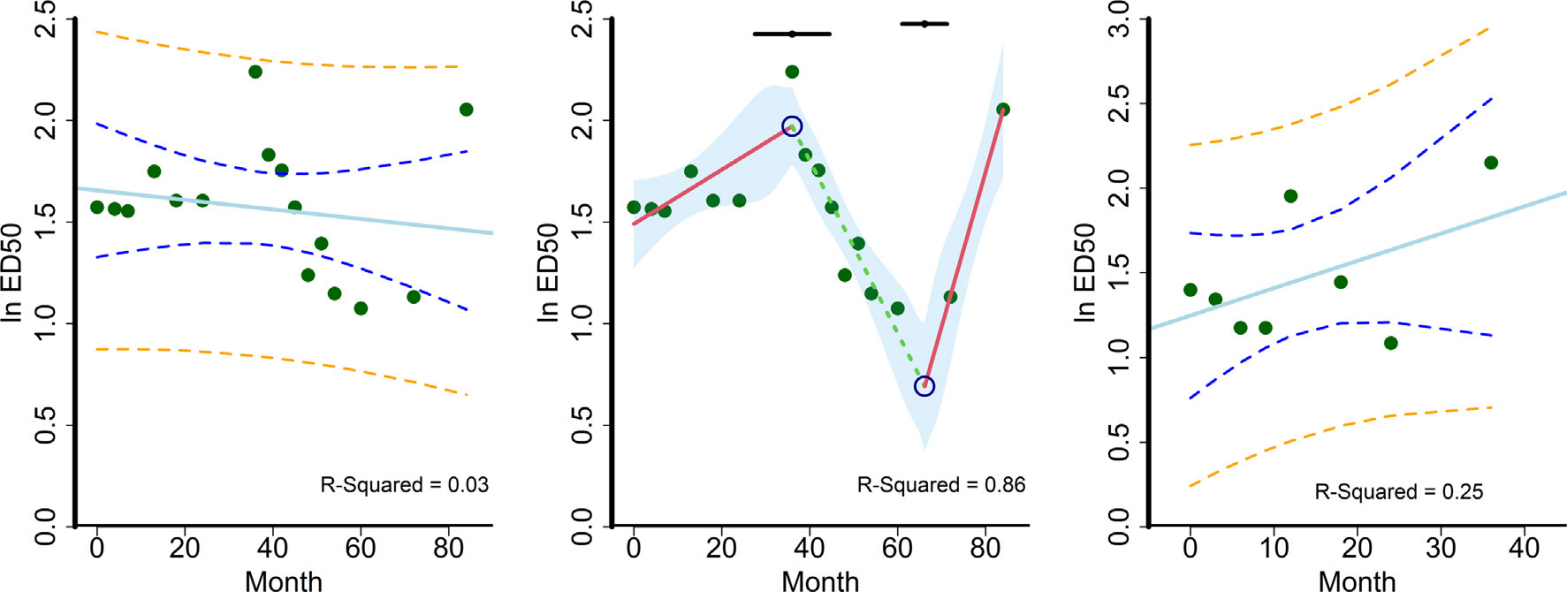
**Linear and joinpoint regressions.** The X-axis represents the testing time points in months post-manufacture and the Y-axis represents the natural log-transformed ED_50_ in µg. The blue solid lines in Panel A and C represent the linear fits for the first (#11-69F-003) and second (#1975) clinical lots, respectively. The green solid circles represent the natural log-transformed ED_50_ in µg at each time point. The blue dotted lines represent the 95% confidence limits and the orange dotted lines represent 95% prediction limits. Panel B shows the change in trend for the first lot (#11-69F-003) in different colors (red for decreasing and green for increasing potency). Break points at 36 and 67 months are shown as blue hollow circles with their 95% confidence intervals shown on the top in black. The shaded area represents the 95% confidence intervals for the joinpoint regression. (For interpretation of the references to color in this figure legend, the reader is referred to the web version of this article.)

The relationship between specific quantitative attributes of potency (i.e., ED_50_) and time is assumed to be linear and, as such, was estimated using simple linear regression as shown in Fig. 3A and C for the first (#11-69F-003) and second (#1975) *Sm*-TSP-2/Al lots, respectively. The low *R^2^* of the linear fit (0.03 for the first lot and 0.25 for the second lot) suggests the inadequacy of assuming a linear relationship between time and potency for *Sm*-TSP-2/Al, with simple visual examination of the data identifying a change of trend at 36 months for the first lot (#11-69F-003). The joinpoint regression analysis for *Sm*-TSP-2/Al (#11-69F-003) showed that breakpoints at 36 and 67 months provided a superior fit to potency than simple linear regression, improving the *R^2^* from 0.03 to 0.86 as shown in Fig. 3B. For the second lot of *Sm*-TSP-2/Al (#1975), simple linear regression also provided an inferior fit, though no breakpoint could be estimated based on the small sample size (n = 8). The decreasing ED_50_ starting at 36 months is indicative of a gain in potency.

### 3.5 Control charting and conforming to formal quality control rules

**Fig. 4:**
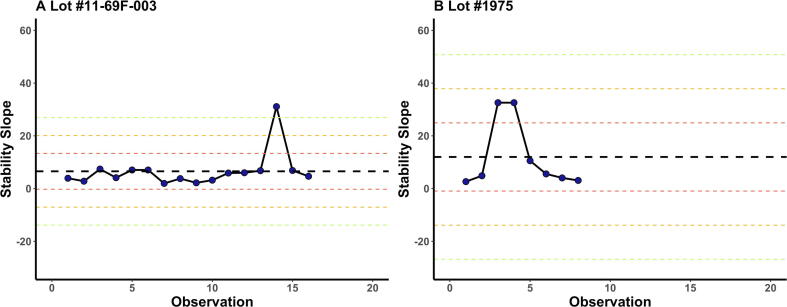
**Levey-Jennings chart of estimated stability slopes for the first lot (#11-69F-003) in Panel A and second lot (#1975) in Panel B.** The X-axis represents the number of testing time points in months post manufacture and the Y-axis represents estimated stability slope from Probit regression model. The black dots show the stability slope at each timepoints in chronological order. Black, red, yellow and green dotted line represent the mean, as well as one, two and three standard deviations to either side of the mean. (For interpretation of the references to color in this figure legend, the reader is referred to the web version of this article.)

Stability slopes *b1* generated from the *probit* regression model at each time point from both lots were plotted on a Levey-Jennings control chart (Fig. 4). There was an upward shift in the stability slopes from the first lot (#11-69F-003) to the second lot (#1975). For the first lot of *Sm*-TSP-2/Al (#11-69F-003), the estimated stability slope of 31 at month 60 exceeded the mean plus three standard deviations (3 s) control limit. In contrast, for the second lot (#1975) of *Sm*-TSP-2/Al, the almost identical estimated stability slope of 32 at months 6 and 9 were within the mean plus two standard deviations (2 s) control limit. Hence, despite straying towards a + 3 s, both lots of *Sm*-TSP-2/Al remained within specification over the entire testing period using the Westgard approach [16].

### 3.6 Bootstrap modeling of potency over time, lot-to-lot comparison, and modeling a sub-potent lot of clinical product

**Fig. 5:**
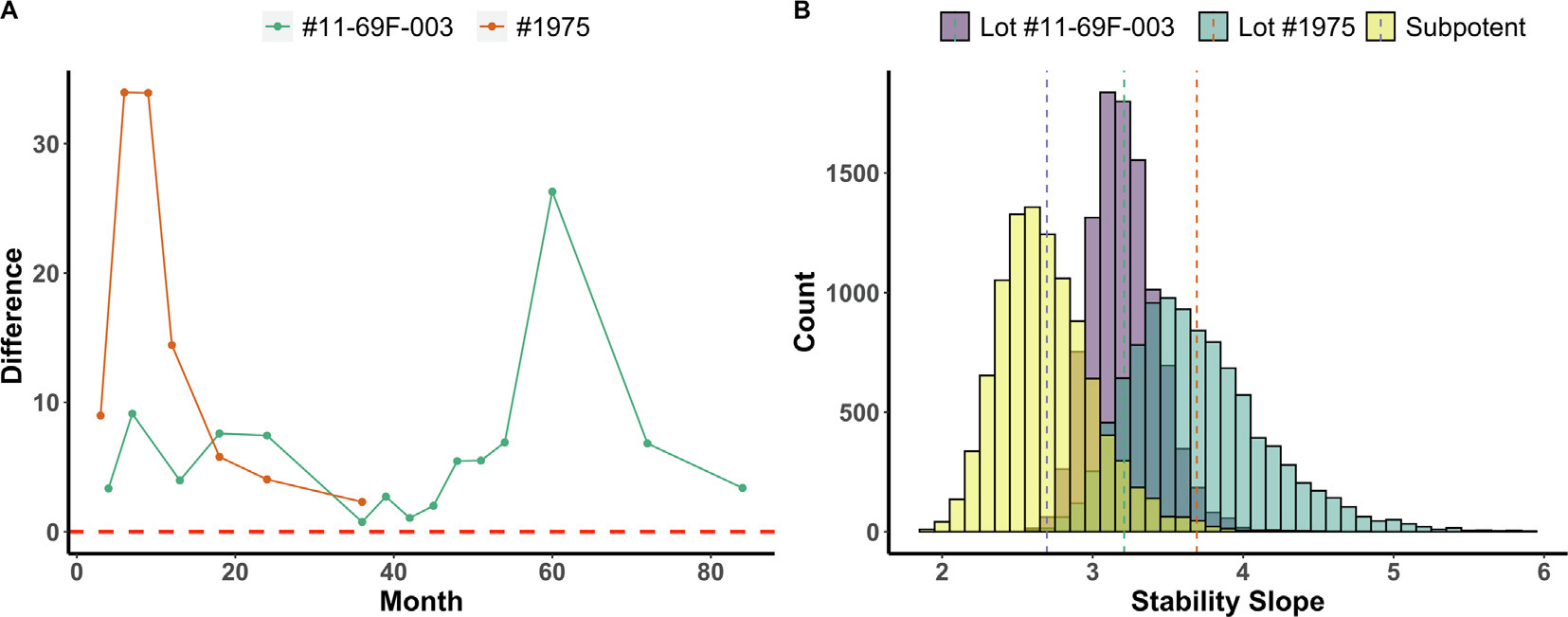
**Bootstrap estimates of stability slopes.** (A) Potency over time: The X-axis represents the testing time points in months post-manufacture and the Y-axis represents the difference between the upper 99% confidence interval of “current” bootstrapped stability slopes and the lower 99% confidence interval of “cumulative” bootstrapped stability slopes. The green and red lines represent the values of difference at each time point for the first (#11-69F-003) and the second (#1975) lots, respectively. The red horizontal dotted line at 0 represents the specification for acceptance that this difference should not be less than 0. **(B)** Lot-to-lot comparison**:** Bootstrap estimates of stability slopes for the first (#11-69F-003), second (#1975) and simulated sub-potent lots. The histogram shows the distribution of 10,000 bootstrap replications of slopes from the first lot (#11-69F-003, in purple), the second lot (#1975, in green), and a simulated sub-potent lot (in yellow). The X-axis represents estimated stability slope from *probit* regression model, and the Y-axis represents the number of stability slope that falls into the corresponding intervals set by X-axis. The three vertical dotted lines, from left to right, represent the estimated mean stability slopes for the simulated sub-potent, the first (#11-69F-003) and the second (#1975) lots, respectively. (For interpretation of the references to color in this figure legend, the reader is referred to the web version of this article.)

Bootstrap replications of stability slopes at each testing timepoint from “current” (in purple) and “cumulative” (in yellow) quantal response data are shown as histograms for the first lot (#11-69F-003) and second lot (#1975) in Fig. S3A and Fig. S3B, respectively. The red vertical dashed lines indicate the 99% confidence interval for the bootstrapped stability slope of “current” response, and the blue vertical dashed lines indicate the 99% confidence interval for the bootstrapped stability slope of “cumulative” response. To better present the results, the mean and 99% confidence intervals for “current” and “cumulative” responses are further shown by the crossbar plot in Fig. S4. If the red crossbar, which represents a “current” bootstrapped stability slope, is below the green crossbar representing “cumulative” bootstrapped stability slope, the lot would be determined to have lost potency. The most straightforward way of determining potency is by calculating the difference between the upper 99% confidence interval of the “current” bootstrapped stability slope and the lower 99% confidence interval of the “cumulative” bootstrapped stability slope (Fig. 5A). If the value is below the horizontal line of 0, the vaccine lot is considered OOS. To make the testing results conform to our current RP paradigm, the 99% confidence interval was chosen instead of the 95% confidence interval. Both lots of *Sm*-TSP-2/Al stayed well within specification over their respective testing periods.

Dose-response relationships were then estimated using bootstrap modeling from the pooled quantal response data across all available time points for the first and second lots, respectively, and the estimated stability slopes *b1* are shown as histograms in Fig. 5B. For the first *Sm*-TSP-2/Al lot (#11-69F-003), the estimated stability slope from the bootstrap simulation was 3.18 (95% confidence interval: 2.67–3.53), and for the second *Sm*-TSP-2/Al lot (#1975), the estimated stability slope was 3.59 (95% confidence interval: 2.42–4.21). The estimated mean slope of the second lot was greater than the estimated mean slope of the first lot. However, the skew of the histogram of the second lot (#1975) to the right suggests that the mean is greater than the median, while the histogram of the first lot (#11-69F-003) is more symmetrical. The overlapping 95% confidence intervals indicate that the stability slopes of the two lots are not significantly different. Lot-to-lot comparison at months 0, 3, 6, and 12 are also presented in Fig. S5. It can be seen that the bootstrapped stability slope histogram evolved into its current shape at month 12. Sub-potent dose–response relationships were then simulated in the same way by pooling quantal response data across those less potent time points (months 36, 39, 42 and 84 for the first lot and month 12 and 36 for the second lot) where the lower 95% confidence limits of the RP were observed to be below or equal to 0.5 as shown in Fig. 1. The estimated slope was 2.64 for simulated less potent dose–response relationships.

## 4 Discussion

As a critical *stability-indicating parameter*, potency is used differently during each phase of vaccine development [2]. During early vaccine development, potency testing is used to determine the initial biological activity of the vaccine and then to ensure maintenance of this activity in early clinical trials [2]. As with several other vaccines against parasitic infections [6], *Sm*-TSP-2/Al does not induce sterilizing immunity in an animal model. Therefore, traditional potency assessment methods [4], which test for protection against lethal challenge infection in immunized animals, are not feasible [13]. Instead, a *serological substitution* method is used in which levels of IgG antibodies detected in the sera of mice immunized with defined doses of *Sm*-TSP-2/Al estimate relative potency at scheduled time points following lot release of the clinical product. This approach has been used for several other parasitic vaccines, including those for malaria [6] and hookworm [12], and is often incorporated into a conventional *compliance* or *single data point* testing approach [10] such as shown in Fig. 1, where potencies of the first two clinical lots of *Sm*-TSP-2/Al were estimated using a *relative potency* (RP) metric.

Several limitations attend potency testing when determined by *serological substitution method* and used in a compliance approach as shown in Fig. 1. In the case of *Sm*-TSP-2/Al, the serological substitution method is an indirect ELISA, in which levels of murine IgG raised against the vaccine antigen (*Sm*-TSP-2) are estimated at pre-defined time point post release. The “intra”- and “inter”- variation of this assay could play a critical role in determining if the potency metric for *Sm*-TSP-2/Al remained within specification (Supplementary Table 1), with an 8.5% risk at each independent time point tested and that the RP of *Sm*-TSP-2/Al could have been OOS due to assay variance alone because of *statistical multiplicity* [10]. The second problem is that, in a compliance approach, the specifications for the potency range, delimited by the lower limit (LL) and upper limit (UL) of potency, are set “*a priori*” at lot release, i.e., in most cases before any actual potency testing has been completed and, as such, are based on little empirical data, making the range an unreliable (if not often ‘arbitrary’) indicator of lot stability over time. These two limitations have led to the assertion that potency testing using a *compliance approach* actually “*discourages*” data collection [10], due to the ever-increasing risk that the clinical product will become OOS, which has significant regulatory ramifications and potential negative impacts on ongoing clinical trials of the investigational product. However, it should be noted that despite these limitations, when assessed using the compliance approach, the first cGMP-manufactured lot of *Sm*-TSP-2/Al (#11-69F-003) remained potent for a remarkable seven years (and counting) after release, while the second cGMP-manufactured lot (#1975) has retained potency for three years (and counting), demonstrating the remarkable stability of this recombinant protein adsorbed to Alhydrogel under typical storage conditions (2–8 °C).

We then tested the potency of both lots of *Sm*-TSP-2/Al using three alternatives to the compliance approach. These were chosen based on three characteristics. The first is that for all three alternative approaches, the potency statistic is less affected by assay variation than the *compliance approach*: e.g., the pre-defined time points tested post-release are not considered separately as in the *compliance model*, so the limitations implicit with multiple testing are not present. The second is that the potency metric is not expected to comply with a “*static”* range of potency limits set prior to initiating potency testing, but can be adjusted based on the data derived from each additional time point tested, thereby becoming more precise with more testing as indicted by either the width of confidence intervals in least-squares regression or the size of the resampling in bootstrap modeling. The third and final factor is that these alternatives to the *compliance approach* can also provide a set of predictive analytics (or trend analysis), which is not possible when only the most recent potency measurement is considered critical as in the *compliance approach*. These include: (a) a least-squares regression analyses of the ED_50_ over time fitted to a first-order linear decay model, and then fitted by a joinpoint regression to indicate when potency deviates from first-order decay; (b) a control charting of stability slopes of the dose–response relationships followed by assessment of conformity to multirule quality control; and (c) bootstrap modeling of potency over time, including a bootstrap simulation of a sub-potent lot of the *Sm*-TSP-2/Al vaccine.

The most robust, and possibly the most intuitive alternative approach to potency testing presented in this manuscript is the least-squares regression model, as set forth by the International Conference of Harmonization [ICH Q13] [19] (Fig. 3). The advantages of a least-squares regression model are numerous, including incorporation of the potency estimates from all available time points, instead of only the most recent as in a *compliance model* (see above). Hence, contrary to a *compliance approach*, the least-squares regression model “*encourages*” data collection since the more potency timepoints measured [10], the more precise the estimation of potency, which can be visualized by the narrowing of the 95% confidence intervals in Fig. 3A and C at 12 months post-release. By estimating the least-squares fit, this approach also enables some anticipation of any future loss or gain of the product’s potency, which is especially important early in clinical development.

As noted by Egan and Schofield [10], a critical drawback to the least-squares regression approach is the requirement of a mathematical model on which to fit the data. In the case of a recombinant protein vaccine, such as *Sm*-TSP-2/Al, the most plausible mathematical model for potency testing is a “*first-order*” or “*linear kinetic decay*” model [10], with the vaccine assumed to degrade in a consistent manner as a function of time, following the *“rate law of the form”* as shown by the solid line in Fig. 3A and C. However, this assumption was not borne out for *Sm*-TSP-2/Al, as the potency of the first lot of the vaccine (#11-69F-003) deviated substantially from first-order kinetics starting at 36 months, when it started in fact to “gain” potency (Fig. 3A and B); the downward trend of the ED_50_, beginning at 36 months, indicates that less vaccine was needed to elicit the same immune response in the bioassay (Fig. 3B). A joinpoint regression constrained to the same post-release timepoints was used to sort through the many possible “segments” (or time points between potency measures) to select the “breakpoint” that best fit such deviations from linear degradation [15].

Similar gains in vaccine potency were observed for another recombinant protein, the *Na*-GST-1 hookworm vaccine antigen, which was also expressed in *P. pastoris* and formulated on Alhydrogel [12]. Potential explanations for this observed gain in potency during storage may include a change in the interaction between the vaccine antigen (*Sm*-TSP-2) and the adjuvant (Al) which might lead to increased potency due to several factors, including (a) auto-extraction of impurities, (b) enhanced binding of the recombinant protein to the adjuvant, or even both of these simultaneously. That is, as the protein adsorbs to the solid surfaces of aluminum adjuvant particles, the surface interactions of the protein might be maximized, potentially changing conformational epitopes and adding stability to the protein. Indeed, shifts or alterations over time in the stability of protein antigens on aluminum particles, both increased and decreased, have been well described [20]. The fit of the joinpoint model illustrates the importance of using such a model to estimate potency for this recombinant protein vaccines.

A second alternative to the *compliance approach* is stability slopes from *probit* analysis graphed onto a Shrewhart chart [16] and monitored by multirule quality control [16]. Fig. 4 shows the stability slopes (*b_1_*) at 84 and 36 months post-release of the first and second cGMP-manufactured lots of *Sm*-TSP-2/Al, respectively. These slopes (*b_1_*) show the rate of change in the probability of seroconversion by log-transformed vaccine dose: with each increasing slope, the probability of seroconversion increases given the same dose level. The benefit of this control charting method is that it monitors the potency properties of the vaccine over time as determined by a series of multiple conventional quality control rules (e.g., the Westgard rules, where the mean plus or minus 1, 2 and 3 standard deviations, i.e., red, yellow and green, as shown by the dotted lines in Fig. 4) with the data continuously updated as new stability slopes become available with each time point tested. The graphical presentation of this method has the obvious advantage of being easy-to-follow along with the rather simple application of the well-established Westgard multirule quality control to evaluate this *stability-indicating parameter*. A limitation of the control chart method is that it does not predict potency trends but merely monitors if the potency metric consistently complies with a multirule convention.

A third and final alternative to a *compliance approach* is the bootstrap modeling of potency as shown in Fig. 5 and Figs. S3–S5. Bootstrap modeling has a long history of estimating the potency of drugs [21]. As a resampling technique “*with replacement”* [22], bootstrapping is a particularly effective statistical approach for potency testing in early clinical development as it can be used even with very little actual data: e.g., time points immediately post-release (e.g., 3, 6, and 12 months). This is because bootstrapping treats any potency data as a proxy for a “*population*” of potency data and draws random samples from this “*population*” [22]. This creates numerous “*resamples*” with their various combinations collectively providing an estimate of the variance in the potency metric, so that hypothesis testing can be performed. Importantly, as the sample size increases (Fig. S5), bootstrapping converges on a more precise sampling distribution with each time point added. As mentioned above, this can address a critical limitation of potency testing during early vaccine clinical development: i.e., the paucity of data on which to base the potency range due to the recent manufacture of the new product. Fig. 5B shows bootstrap estimates of stability slopes for both manufactured lots (#11-69F-003 and #1975) of *Sm*-TSP-2/Al, with each histogram representing the distribution of 10,000 bootstrap replications of stability slopes of the two lots. The simulated sub-potent lot was derived using the six lowest *relative potency* levels of *Sm*-TSP-2/Al.

**Fig. 6:**
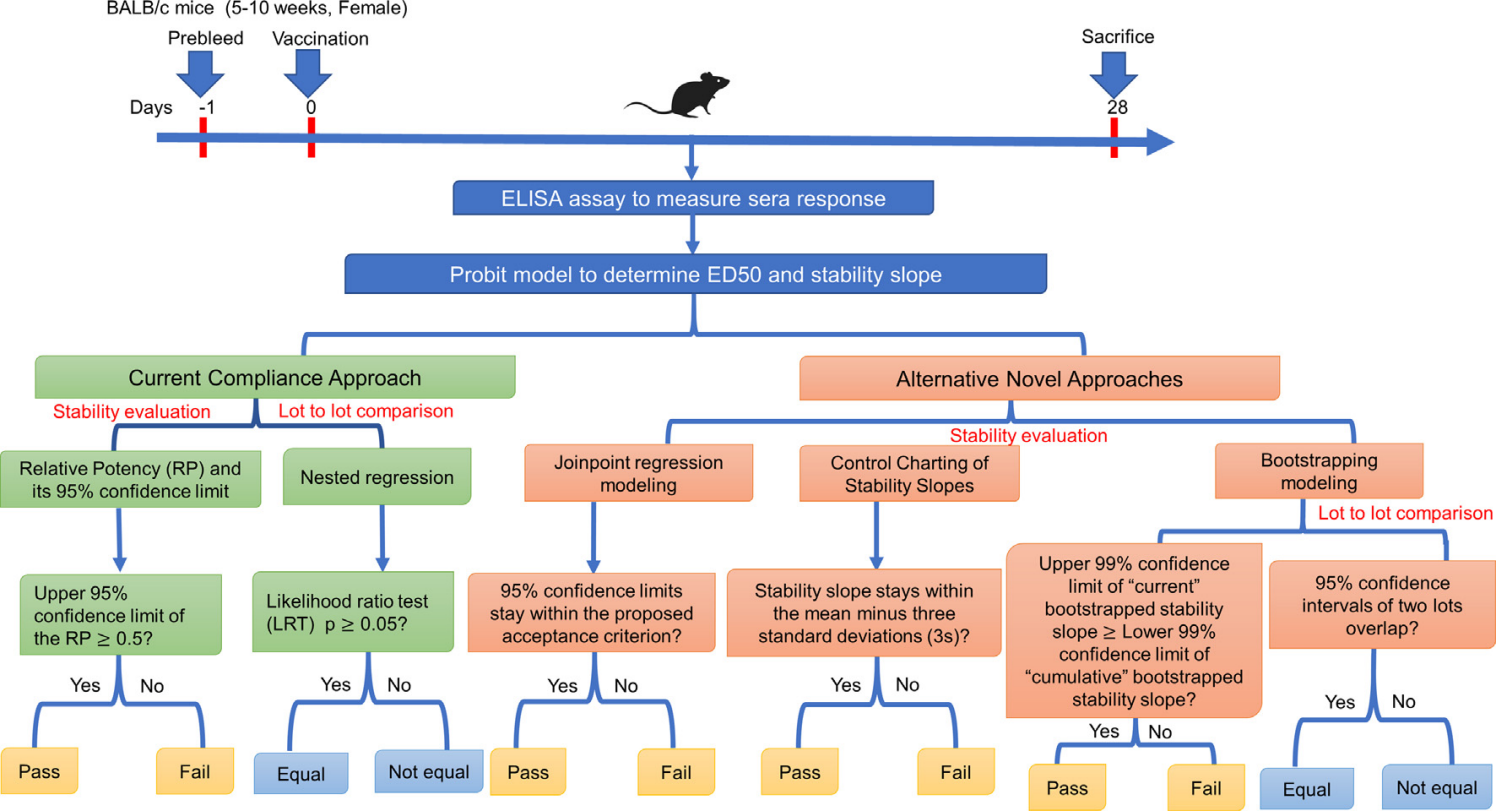
Process scheme summarizing potency testing approaches of the *Sm*-TSP-2 vaccine for both clinical lots (#11-69F-003 and #1975).

The potency testing approaches presented here add to the literature on this critical indicator of vaccine stability early in vaccine clinical development by providing alternatives to a conventional *compliance model*. A process scheme summarizing potency testing approaches for both clinical lots is shown in Fig. 6. It should be noted that, in all four approaches to potency testing presented, the first and the second GMP-manufactured lots of *Sm*-TSP-2/Al remained potent for a remarkable 84 and 36 months, respectively, when stored at 2-8˚C, indicating the robust stability of this recombinant protein vaccine.

## Funding

This work was supported in part by intramural funding from Texas Children’s Center for Vaccine Development at Baylor College of Medicine.

## CRediT authorship contribution statement

**Guangzhao Li:** Conceptualization, Methodology, Software, Formal analysis, Investigation, Writing - original draft, Writing - review & editing, Visualization, Data curation, Validation, Project administration. **Lara Hoeweler:** Investigation, Data curation, Writing - review & editing, Project administration, Validation. **Brian Keegan:** Investigation, Data curation, Writing - review & editing, Project administration, Validation. **Jin Peng:** Investigation, Data curation, Project administration, Validation. **Larissa Scholte:** Writing - review & editing, Validation. **Peter Hotez:** Resources, Writing - review & editing, Funding acquisition, Validation. **Maria Elena Bottazzi:** Resources, Writing - review & editing, Funding acquisition, Supervision, Validation. **David Diemert:** Writing - review & editing, Validation. **Jeffrey Bethony:** Conceptualization, Validation, Formal analysis, Investigation, Resources, Writing - original draft, Writing - review & editing, Visualization, Supervision.

## Declaration of Competing Interest

The authors declare that they have no known competing financial interests or personal relationships that could have appeared to influence the work reported in this paper.
