# Supplementary material for: Potency testing for a recombinant protein vaccine early in clinical development: Lessons from the Schistosoma mansoni Tetraspanin 2 vaccine
Source: Vaccine X. 2021 Jun 6;8:100100. doi: 10.1016/j.jvacx.2021.100100 (PMC8209742; doi:10.1016/j.jvacx.2021.100100)
Supplement: Supplementary data 1 [file mmc1.docx]

**Supplementary Material**

1. **Supplementary Texts**
2. Supplementary text 1: *Schistosoma mansoni (Sm)* Tetraspanin-2 vaccine formulated on Alhydrogel (*Sm*-TSP-2/Al)
3. Supplementary text 2: Potency Testing Program
4. Supplementary text 3: Relative potency (RP) derivation and application
5. Supplementary text 4: Nested regression for lot-to-lot variation
6. Supplementary text 5: Least-squares regression analysis followed by joinpoint regression

a. Derivation and application of first-order decay kinetics model

b. Maximum-likelihood (ML) estimation

6) Supplementary text 6: Application of Westgard rules to control chart

7) Supplementary text 7: Bootstrap estimation

a. The approximate bootstrap confidence intervals of stability slope b_1_ for the “current” timepoint respondence and the “cumulative” timepoints respondence

b. The approximate bootstrap confidence intervals of stability slope b_1_ for the first lot and the second lot

1. **Supplementary Figures**
2. **Supplementary Tables**
3. **References**

**Supplementary Text 1**: ***Schistosoma mansoni (Sm)* Tetraspanin-2 vaccine formulated on Alhydrogel (*Sm*-TSP-2/Al)**

*Sm*-TSP-2/Al is the recombinant *Schistosoma mansoni* tetraspanin-2 protein (*Sm*-TSP-2) formulated on Alhydrogel^®^ being developed for use in children and adults as a prophylactic vaccine against infection or reinfection from intestinal/hepatic schistosomiasis. The first clinical lot (#11-69F-003) was formulated by Aeras (Rockville, MD) at a concentration of 0.1 mg/mL *Sm*-TSP-2 with 0.8 mg/mL of Alhydrogel^®^ in a sucrose/imidazole/Phosphate buffer (15% sucrose, 10mM imidazole, 2 mM Phosphate, pH 7.4) in December 2011. The second clinical lot (#1975) was manufactured by the Walter Reed Army Institute of Medicine (WRAIR, Silver Spring, MD) using the same formulation conditions in February 2016. Both lots of *Sm*-TSP-2/Al were manufactured in compliance with cGMP regulations and subsequently stored in temperature-monitored refrigerators at 2-8°C.

**Supplementary Text 2: Potency Testing Program**

The *Sm*-TSP-2/Al potency testing program uses a standard calibration curve (SCC) to interpolate levels of immunoglobulin G (IgG) against *Sm*-TSP-2 in an indirect ELISA format. In brief, a standard reference serum (SRS) of murine IgG against *Sm*-TSP-2 was generated from the pooled sera of 40 BALB/c mice vaccinated with 9.33μg of recombinant *Sm*-TSP-2 (clinical Drug Substance) and 74.64μg of Alhydrogel^®^ by intraperitoneal (IP) injection. Female BALB/c mice purchased from a certified vendor were used for these experiments. All animals were treated in accordance with the PHS Guide for the Care and Use of Laboratory Animals 8th edition under a Baylor College of Medicine Institutional Animal Care and Use Committee approved protocol.

The starting dilution of the SRS is added to a microtiter plate in duplicate and assigned a value of 1000 arbitrary units (AU) and then added in doubling dilutions in duplicate (2X) with a similar multiplication factor applied to the AUs, with the resulting optical densities (OD) at 492nm plotted against the dilution factor. The data are described by the following four-parameter logistic-log function:

*y*= D +$\frac{(A-D)}{1+{(\frac{X}{C})}^{B}}$

where *X* is the dilution in AUs, *y* is the measured response in OD, *A* represents the upper asymptote, *B* is the slope of the curve, *C* represents the theoretical dilution in AUs that produce a response equal to 50% of the maximum responses, and *D* is the lower asymptote.

The design of the potency tests conducted at each time point is shown in Table 1. BALB/c mice were divided into 11 dose groups of 10 mice. The BALB/c mice in Group 1 were immunized with 400µg of Alhydrogel^®^ alone; the BALB/c mice in Group 2 with 50µg of *Sm*-TSP-2 alone. BALB/c mice in Groups 3 to 10 were immunized in 1.75-fold increasing fractional doses of the vaccine starting from 0.99μg to 50μg of *Sm*-TSP-2/Al with the ratio of the dose of *Sm*-TSP-2/Al held constant at 0.125. This *Sm*-TSP-2/Al potency assay uses a “*quantal”*’ response method in which a serum sample is assigned a status of seropositivity if it has a level of IgG against *Sm*-TSP-2 that is above a “*Reactivity Threshold*” (RT). The data of resulting percent responders in Groups 3-10 were analyzed using the *PROBIT* function of SAS^®^ (Statistical Analysis System) software, version 9.4 to estimate the theoretical dose in µg that would produce seroconversion in 50% of the mice (ED_50_).

**Supplementary Text 3**: **Relative potency (RP) derivation and application**

Step 1: The number of mice in each group that seroconverted at a testing timepoint was determined based on cutoff value RT as described in the potency testing program section (see Supplementary Text 2).

Step 2: The proportions of responders are entered into a working table using the method introduced by the European Pharmacopoeia [1]. An example working table is shown below, where the table entitled “Standard” represents responders at release and table entitled “Test” represents responders at the current testing timepoint.

Step 3: Calculation is repeated by updating common slope b and Y = a + bx until convergence is achieved, i.e., the difference of Y between two working tables in sequence has become small (less than 10^-8^).

Step 4: RP and its confidence limits can then be obtained using values from the last working table. Details of the calculation of confidence limits can be found in the European Pharmacopoeia [1].

Step 5: RP for each testing timepoint is calculated and plotted as point ranges as shown in Fig. 1. If the upper confidence limit of RP is below 0.5, the lot is out of specification.

**Supplementary Text 4:** Example SAS codes comparing the first and second lots at release are presented below; codes are adapted from Oris and Bailer [14]:

*data comp;
input prl $ Dose resp n;
i = (prl = 'new');
lDose=log10(Dose);
id=i * lDose;
cards;
OLD 50 10 10
OLD 28.57 10 10
OLD 16.33 10 10
OLD 9.33 9 10
OLD 5.33 4 10
OLD 3.05 1 10
OLD 1.74 1 10
OLD 0.99 1 10
new 50 9 10
new 28.57 10 10
new 16.33 9 10
new 9.33 10 10
new 5.33 10 10
new 3.05 3 10
new 1.74 0 10
new 0.99 0 10
;*

*proc print data=comp;
run;*

*proc probit COVOUT lackfit
outest = out2;
model resp /n= lDose i id/itprint inversecl corrb COVB;
      output out=B p=Prob std=std xbeta=xbeta;
   predpplot CFIT=blue WFIT=2;
title ‘FULL MODEL - - All Parameters Specified’;
run;*

*proc probit COVOUT lackfit
outest = out3;
model resp /n= lDose/itprint inversecl corrb COVB;
      output out=B p=Prob std=std xbeta=xbeta;
   predpplot CFIT=blue WFIT=2;
title ‘Beta2=Beta3=0? (if reject, then intercepts
and/or slopes differ)’;
run;*

**Supplementary Text 5:**

a. Derivation and application of first-order decay kinetics model

Step 1: The potency parameter ED_50_ can be described by the following equation

ED_50_(*t*) = ED_50_(0) $e^{(-kt)}$

Where,

ED_50_(*t*) is the potency as measured by ED_50_ at time point t,

ED_50_(0) is the potency as measured by ED_50_ at release,

K is the first-order rate constant representing potency loss rate

Step 2: Taking logarithms on both sides yields

lnED_50_(*t*) = lnED_50_(0) – kt

Step 3: Using *Sm*-TSP-2 data, the ordinary least-squares method is employed to obtain the estimates of slope (rate constant k), so that the residual sum of squares (the square of the vertical distance between the fitted straight line and the actual ED_50_ values) are minimized.

Step 4: The estimated line is drawn and the fit of this regression line to the actual data is measured by *R^2^*.

Step 5: If lack of fit (change of trend) is evident, a joinpoint regression is fitted as an alternative next step.

b. Maximum-likelihood (ML) estimation

The maximum-likelihood (ML) estimation approach of Muggeo [15] yields the following steps:

Step 1: According to first-order Taylor’s expansion, the following equation assuming an initial break-point value ${}^{(0)}$ is obtained

(t-τ)^+^ = (t-${}^{(0)}$)+(τ-${}^{(0)}$)(-1) *Ι* ($t$>${}^{(0)}$)

Where *Ι* is the indicator variable and (-1) *Ι* ($t$>${}^{(0)}$) is the first derivative of (t-τ)^+^ evaluated at ${}^{(0)}$.

Step 2: Let β = α (τ-${}^{(0)}$), the right side of

lnED_50_(*t*) = lnED_50_(0) - kt *+ α* (t-τ)^+^

can now be estimated by the following

lnED50(0) - kt + α (t-${}^{(0)}$) + β (-1) Ι ($t$>${}^{(0)}$)

Step 3: Break-point approximation is improved at every iteration by updating parameter τ with τ = $\frac{}{}$ + ${}^{(0)}$.

Step 4: Repeat steps 1-3 until convergence is achieved.

**Supplementary Text 6:** Application of Westgard rules to control chart

Step 1: Stability slope b_1_ is generated at each time point using *PROBIT* function of SAS^®^.

Step 2: Available stability slopes are plotted using Levy-Jennings charts, where means and one to three standard deviations (SDs) are calculated and plotted as presented in Fig. 4.

Step 3: Subsequent stability slopes are entered into the control chart.

Step 4: The lot is considered to be out of specification if the stability slope exceeds the mean minus three standard deviations.

**Supplementary Text 7:**

1. The approximate bootstrap confidence intervals of stability slope b_1_ for the “current” timepoint respondence and the “cumulative” timepoints respondence were calculated as follows:

Step 1: At timepoint n, individual quantal response of each mouse from all n time points of a single lot are pooled together to form sample {D*_i_*, S*_i_*}, where D is dosage level, and S is response (1 for seropositive and 0 for seronegative).

Step 2: Stability slope b_1_ is estimated from sample {D*_i_*, S*_i_*} using the function glm() in R software (version 4.0.0). This is accomplished by specifying the *probit* link function under the argument *family*.

Step 3: Sample with replacement of the same sample size as the original data to form {D*_i_**, S*_i_**}.

Step 4: b_1_* is the stability slope computed from the resampled data {D*_i_**, S*_i_**}.

Step 5: Repeat steps 3-4 for B = 10,000 times, and thus a sample of n=B is created for b_1_. This is accomplished by utilizing the boot() function from R package “boot”[18; 17]. The empirical distribution of b_1_ is presented as a histogram labeled as “cumulative” in Fig. S3. 99% basic confidence interval for stability slope b_1_ is obtained using the boot.ci() function.

Step 6: Repeat steps 1-5 using response data at time point n only of this lot to obtain the empirical distribution and 99% basic confidence interval of b_1_. The empirical distribution of b_1_ is presented as a histogram labeled as “current” in Fig. S3.

Step 7: The difference between the upper 99% confidence interval of the “current” bootstrapped stability slope and the lower 99% confidence interval of the “cumulative” bootstrapped stability slope at each testing timepoint is calculated and presented in Fig. 5A; if this value goes below 0, the lot becomes out of specification.

1. The approximate bootstrap confidence intervals of stability slope b_1_ for the first lot and the second lot were calculated as follows:

Step 1: Individual quantal response of each mouse from all available time points of the first lot (#11-69F-003) are pooled together to form sample {D*_i_*, S*_i_*}, where D is dosage level, and S is response (1 for seropositive and 0 for seronegative).

Step 2: Stability slope b_1_ is estimated from sample {D*_i_*, S*_i_*} using the function glm() in R software (version 4.0.0). This is accomplished by specifying the *probit* link function under the argument *family*.

Step 3: Sample with replacement of the same sample size as the original data to form {D*_i_**, S*_i_**}.

Step 4: b_1_* is the stability slope computed from the resampled data {D*_i_**, S*_i_**}.

Step 5: Repeat steps 3-4 for B = 10,000 times, and thus a sample of n=B is created for b_1_. This is accomplished by utilizing the boot() function from R package “boot”[17; 18]. The empirical distribution of b_1_ is presented as a histogram as shown in Fig. 5B. The 95% confidence interval for stability slope b_1_ is obtained using the boot.ci() function.

Step 6: Repeat steps 1-5 using pooled data from all available time points of the second lot (#1975) to obtain the empirical distribution and 95% confidence interval of b_1_.

Step 7: Repeat steps 1-5 using pooled data from less potent time points where lower 95% confidence limits of RP crossed 0.5 as presented in Fig. 1. Empirical distribution and 95% confidence interval of b_1_ for the potential sub-potent lot are generated accordingly.

**SUPPLEMENTARY FIGURES**

**
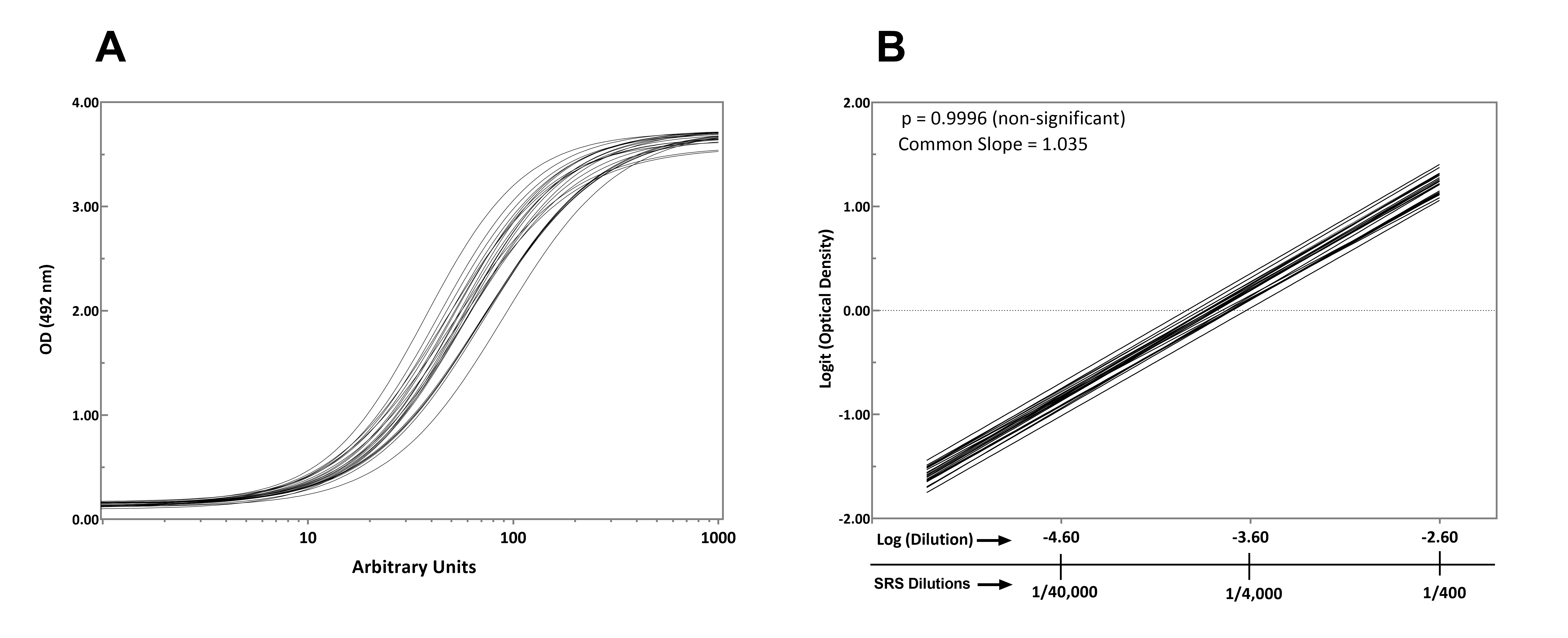
Fig. S1. Standard Calibration Curves and Parallelism Testing**

**(A)** Standard Calibration Curves: Twenty-four standard calibration curves (SCCs) generated at different time points were plotted along a four-parameter logistic log scale, where the X-axis represents the log of the dilution of SRS in AU and the Y-axis its Optical Density (OD) of 492nm. The lines represent the anti-*Sm*-TSP-2 IgG ELISAs performed at 16 time points over 84 months for the first lot (#11-69F-003) and 8 time points over 36 months for the second lot (#1975). The term SRS refers to the Standard Reference Serum and the term AU refers to Arbitrary Units. **(B)** Parallelism Test: Linearization of the 24 SCCs shown in panel (A) using a logit-log scale. The X-axis represents the log of the dilution and the Y-axis represents the fully specified logit of OD_492nm_. Tests of parallelism were performed using an ANOVA test, which indicated no significant departure from parallelism (p = 0.9996).

**
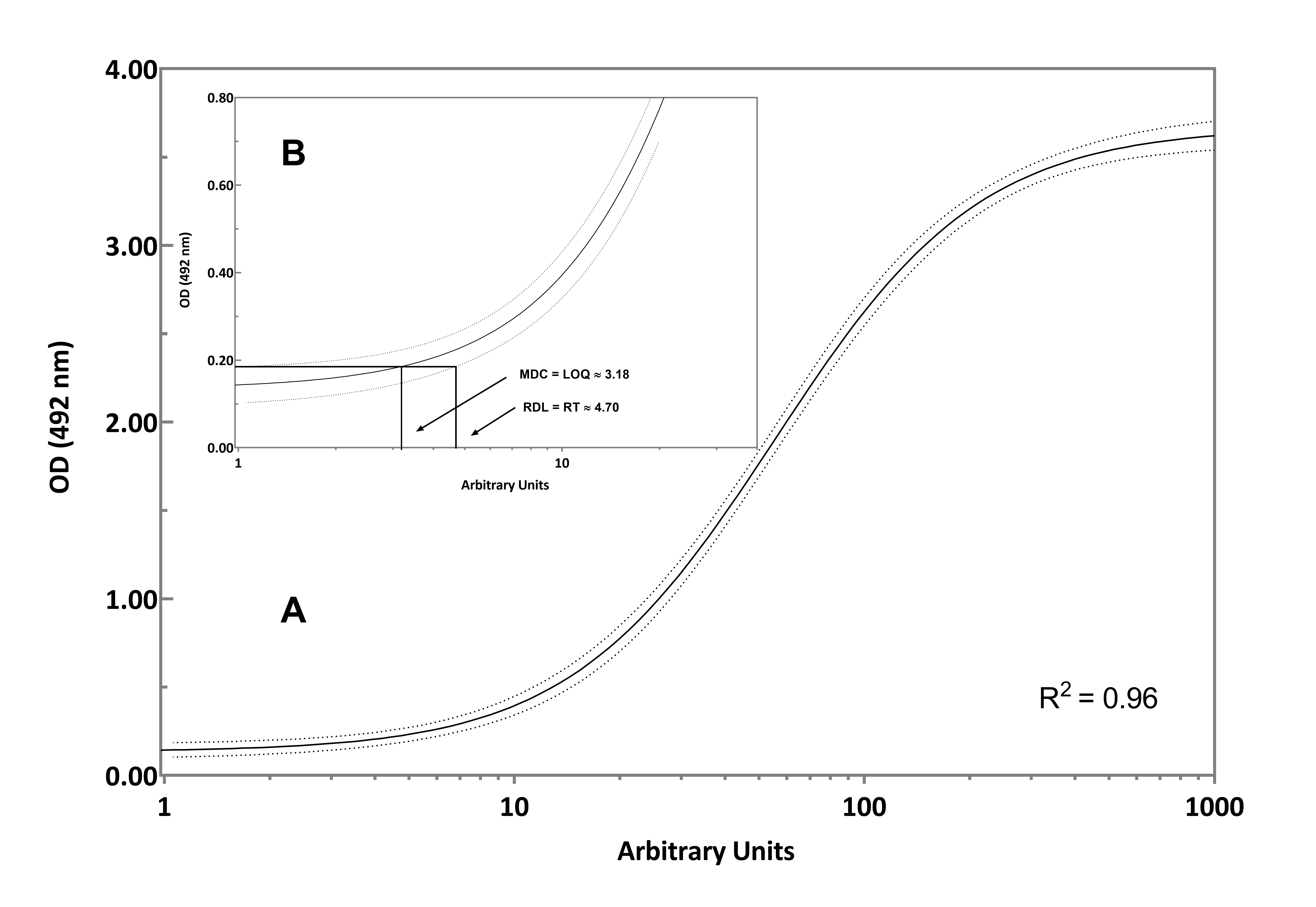
**

**Fig. S2. Global Standard Calibration Curves (GSCC), Reactivity Thresholds (RT), and the Limits of Quantitation (LOQ).**

1. Global Standard Calibration Curves: A global SCC (GSCC) with the 95% confidence interval generated by combining the twenty-four curves shown in Fig. S1A. The X-axis represents the log of the dilution of SRS in AU and the Y-axis is the Optical Density (OD) at 492nm. The term AU refers to Arbitrary Units.
2. RT and LOQ: Reactivity threshold and limit of quantitation as determined from the GSCC shown in panel (A). The X-axis represents the log of the dilution of SRS in AU and the Y-axis is Optical Density (OD) of 492nm.


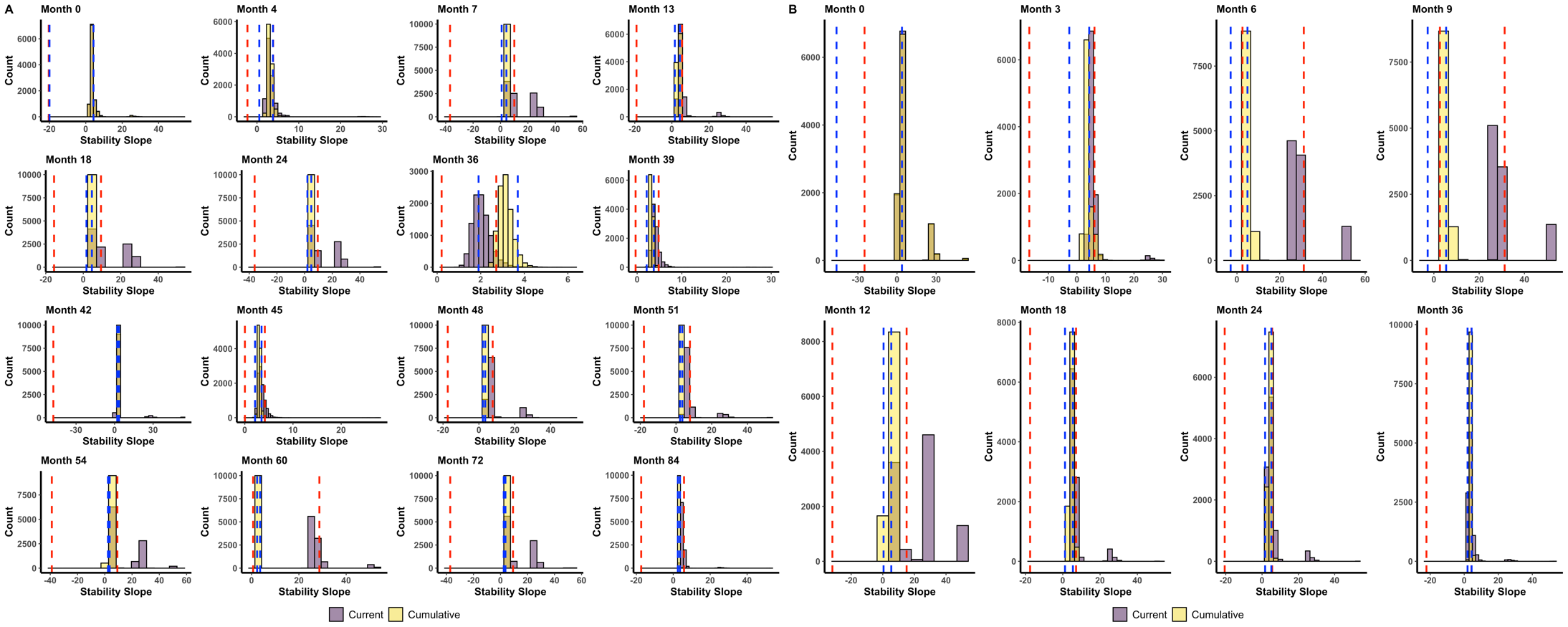


**Fig. S3. Bootstrap estimates of stability slopes.** Bootstrap estimates of stability slopes for the first lot (#11-69F-003) in Panel A and second lot (#1975) in Panel B. Histogram shows the distribution of 10,000 bootstrap replications of slopes at each transient testing timepoint from “current” (in purple) and “cumulative” (in yellow) response data. X-axis represents estimated stability slope from *probit* regression model, and the Y-axis represents the number of stability slopes that fall into the corresponding intervals set by the X-axis. Red vertical dashed lines indicate the 99% confidence interval for the bootstrapped stability slope of “current” response data, and blue vertical dashed lines indicate the 99% confidence interval for the bootstrapped stability slope of “cumulative” response data.


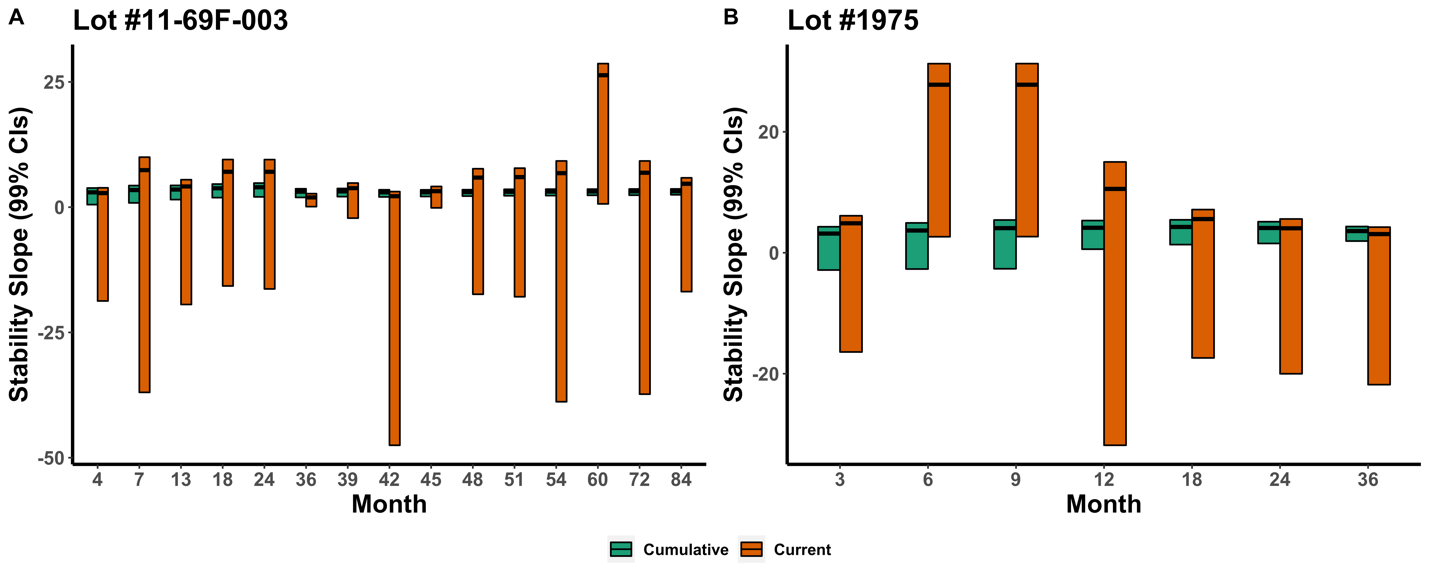


**Fig. S4. Crossbar of the mean and 99% confidence intervals for “current” and “cumulative” responses.** The X-axis represent the testing time points in months post manufacture and the Y-axis represents estimated stability slope from *probit* regression model and its 99% confidence limits. The green bar represents the “cumulative” and the red bar represents the “current”, where "current" refers to the bootstrapped stability slope generated using response data at current timepoint *n*, and “cumulative” refers to the bootstrapped stability slope generated using the pooled response data until timepoint *n*. If the red crossbar representing “current” bootstrapped stability slope is below the green crossbar representing “cumulative” bootstrapped stability slope, the lot is determined to have lost potency. Panels A and B are for the first (#11-69F-003) lot and second (#1975) lot, respectively.

**
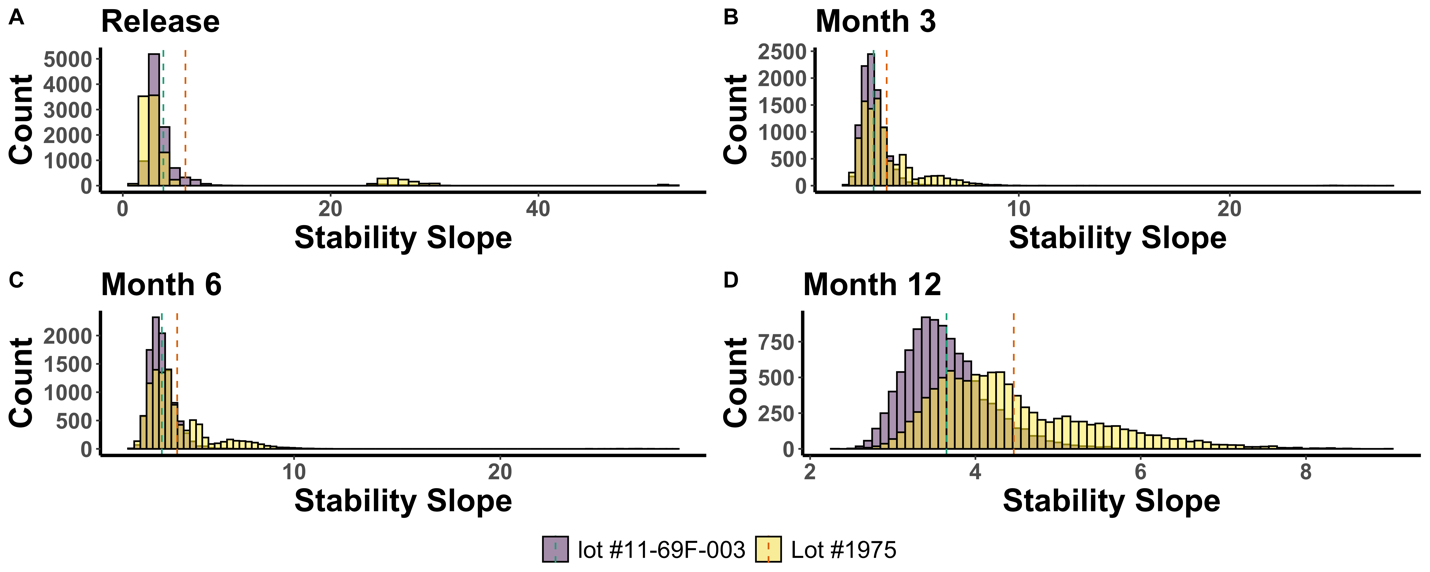
Fig. S5. Bootstrap estimates of stability slopes for the first (#11-69F-003) and second (#1975) lots at transient timepoints months 0, 3, 6, and 12**. The histogram shows the distribution of 10,000 bootstrap replications of slopes from the first (#11-69F-003) lot (in purple) and the second (#1975) lot (in yellow). The X-axis represents estimated stability slope from *probit* regression model, and the Y-axis represents the number of stability slopes that fall into the corresponding intervals set by the X-axis. The vertical dotted lines represent the estimated mean stability slope for the first (#11-69F-003) lot in green and second (#1975) lot in red, respectively. Panels A, B, C and D show results at release, 3^rd^, 6^th^ and 12^th^ month, respectively.

| **Supplementary Table 1: Impact of statistical multiplicity on likelihood of within and out-of-specification results for potency testing using a compliance approach in which each potency test is considered independently.** | | | |
| --- | --- | --- | --- |
| No. of measurements | Month | Probability^1^  Within Specification | Probability^2^  Out of Specification |
| 1 | 0 | 91.5% | 8.5% |
| 2 | 3 | 83.7% | 16.3% |
| 3 | 6 | 76.6% | 23.4% |
| 4 | 9 | 70.1% | 29.9% |
| 5 | 12 | 64.1% | 35.9% |
| 6 | 18 | 58.7% | 41.3% |
| 7 | 24 | 53.7% | 46.3% |
| 8 | 36 | 49.1% | 50.9% |
| 9 | 48 | 45.0% | 55.0% |
| 10 | 60 | 41.1% | 58.9% |
| 11 | 72 | 37.6% | 62.4% |
| ^1^ Probability that all measurements fall within specification  ^2^ Probability that one or more tests erroneously fall out of specification (OOS) | | | |

| **Supplementary Table 2. Number of responders by formulation group and potency time point for the *Sm*-TSP-2/Al potency assay.** | | | | | | | | | | | | | | | | |
| --- | --- | --- | --- | --- | --- | --- | --- | --- | --- | --- | --- | --- | --- | --- | --- | --- |
|  | Number of Positive Responders by Month^a^ | | | | | | | | | | | | | |  |  |
| Aeras Lot #11-69F-003 | | | | | | | | | | | | | | | | |
| Group | 0 | 4 | 7 | 13 | 18 | 24 | 36 | 39 | 42 | 45 | 48 | 51 | 54 | 60 | 72 | 84 |
| 1 | 0 | 0 | 0 | 0 | 0 | 0 | 0 | 0 | 0 | 0 | 0 | 0 | 0 | 0 | 0 | 0 |
| 2 | 1 | 0 | 0 | 0 | 0^b^ | 0 | 0 | 0 | 0 | 0 | 0 | 0 | 0 | 0 | 0 | 0 |
| 3 | 10 | 10 | 10 | 10 | 10 | 10 | 8 | 10 | 7 | 10 | 10 | 10 | 10 | 10 | 10 | 10 |
| 4 | 10 | 9 | 10 | 10 | 10 | 10 | 7 | 10 | 10 | 10 | 10 | 10 | 10 | 10 | 10 | 10 |
| 5 | 10 | 10 | 10 | 9 | 10 | 10 | 9 | 10 | 10 | 9 | 10 | 10 | 10 | 10 | 10 | 10 |
| 6 | 9 | 10 | 10 | 9 | 10 | 10 | 7 | 6 | 10 | 9 | 10 | 10 | 10 | 10 | 10 | 4 |
| 7 | 4 | 4 | 6 | 5 | 5 | 5 | 4 | 4 | 8 | 4 | 8 | 7 | 10 | 10 | 9 | 4 |
| 8 | 1 | 2 | 1 | 1 | 1 | 1 | 1 | 2 | 0 | 5 | 5 | 3 | 3 | 7 | 6 | 0 |
| 9 | 1 | 2 | 0 | 0 | 0 | 0 | 0 | 0 | 0 | 0 | 0 | 0 | 1 | 0 | 0 | 0 |
| 10 | 1 | 0 | 0 | 0 | 0 | 0 | 0 | 0 | 0 | 0 | 0 | 0 | 0 | 0 | 0 | 0 |
| 11 | 9 | 8 | 10 | 10 | 6 | 4 | 7 | 5 | 9 | 10 | 10 | 10 | 10 | 10 | 6 | 5 |
| WRAIR Lot #1975 | | | | | | | | | | | | | | | |  |
| Group | 0 | 3 | 6 | 9 | 12 | 18 | 24 | 36 |  |  |  |  |  |  |  |  |
| 1 | 0 | 0 | 0 | 0 | 0 | 0 | 0 | 0 |  |  |  |  |  |  |  |  |
| 2 | 0 | 0 | 0 | 0 | 0 | 0 | 0 | 0 |  |  |  |  |  |  |  |  |
| 3 | 9 | 10 | 10 | 10 | 10 | 10 | 10 | 9 |  |  |  |  |  |  |  |  |
| 4 | 10 | 10 | 10 | 10 | 10 | 10 | 10 | 10 |  |  |  |  |  |  |  |  |
| 5 | 9 | 10 | 10 | 10 | 10 | 10 | 10 | 9 |  |  |  |  |  |  |  |  |
| 6 | 10 | 10 | 10 | 10 | 9 | 10 | 10 | 7 |  |  |  |  |  |  |  |  |
| 7 | 10 | 6 | 10 | 10 | 1 | 6 | 9 | 1 |  |  |  |  |  |  |  |  |
| 8 | 3 | 5 | 2 | 2 | 0 | 3 | 4 | 1 |  |  |  |  |  |  |  |  |
| 9 | 0 | 0 | 0 | 0 | 0 | 0 | 1 | 0 |  |  |  |  |  |  |  |  |
| 10 | 0 | 0 | 0 | 0 | 0 | 0 | 1 | 0 |  |  |  |  |  |  |  |  |
| 11 | 10 | 7 | 10 | 10 | 10 | 10 | 10 | 10 |  |  |  |  |  |  |  |  |
| ^a^ The number of BALB/c mice seroconverting by dose group, with ten mice per group. | | | | | | | | | | | | | | | |  |
| ^b^ Five mice dead in the group. | | | | | | | | | | | | | | | |  |

| **Supplementary Table 3. Dose-response relationship comparisons between two lots at each testing timepoint** | | | | | |
| --- | --- | --- | --- | --- | --- |
| Month | Full Model | | β_2_=β_3_=0? | LRT* | P-value |
| **0** | 95.89 | | 96.82 | 0.93 | 0.628 |
| **3** | 82.56 | | 87.09 | 4.53 | 0.104 |
| **6** | 30.44 | | 37.09 | 6.65 | **0.036** |
| **12** | 48.62 | | 55.78 | 7.16 | **0.028** |
| **18** | 48.91 | | 50.45 | 1.54 | 0.464 |
| **24** | 57.27 | | 68.43 | 11.17 | **0.004** |
| *****Likelihood ratio test | |  |  |  |  |

**References**

European Pharmacopoeia Commission. (2008). Statistical analysis of results of biological assays and tests. *European Pharmacopoeia*, 571-600.
